# Metacognition and epistemic injustice in schizophrenia

**DOI:** 10.3389/fpsyt.2024.1525178

**Published:** 2025-01-08

**Authors:** Narmin Abasova, Anita Pacholik-Żuromska

**Affiliations:** Department of Cognitive Science, Institute of Information and Communication Research, Nicolaus Copernicus University in Toruń, Toruń, Poland

**Keywords:** metacognition, schizophrenia, epistemic injustice, first person authority, metacognitive therapy

## Introduction

Consider a real situation: During an interview, a patient with schizophrenia, after experiencing strong hallucinations, asked a doctor, “Doctor, I know it’s impossible. But … is it possible that I saw x? Could x really happen?” To protect the patient’s privacy, the specific content of the hallucination is omitted. Also, our focus is not on the experience itself but on the doctor’s response: “I believe that you experienced x. I believe you saw x. But x was not real.” This response exemplifies a respectful acknowledgment of the patient’s first-person authority (FPA). Below, we explore why this example is inspiring for the treatment of schizophrenia. To this end, we will first discuss the relation between FPA and metacognition. This is essential to demonstrate why metacognitive therapies should be a focus in schizophrenia treatment. Integrating metacognition into schizophrenia therapy will then constitute the next point of our considerations. Finally, we will discuss the dangers of undermining FPA for therapeutic success, while also addressing the risks of overemphasizing FPA at the expense of necessary medical oversight.

## FPA in metacognition

FPA accompanies metacognition, defined as thinking about one’s own thoughts and cognitions (1–3). In philosophical terms, it is called self-knowledge—i.e., the subject’s first-person knowledge of their own mental states, to which they have privileged access (4–6). This refers to propositional knowledge expressed in beliefs: “I believe (feel) that…”, “This is my belief, experience, intention, etc. about…”. The concept of knowledge is crucial because it lies at the core of the authority of first-person judgments and is legitimated by direct access to one’s own mental states (7). Beliefs concerning oneself – *de se* beliefs (8) are often considered as being infallible, incorrigible and self-intimating (5, p. 91). The difference between self-knowledge and metacognition is that the former concerns only intentional, conscious states, whereas the latter can be rooted in bodily information processing, such as proprioception (9) and interoception (10), which provide information about the body and serve for self-other distinctions (11). Thus, metacognition can be conceptual—pertaining to one’s knowledge about their thoughts and experiences—or non-conceptual, in the form of bodily self-awareness. In this paper, we refer to metacognition as a subject’s conscious first-person beliefs about oneself.

The first-person perspective underlying metacognition gives the subject a strong sense of certainty about their experiences and thoughts, i.e., a sense of FPA toward themselves, although this can be challenged by empirical findings, such as self-illusions, for example the full-body illusion or body-swap illusion (12, 13). Phantom pains and hallucinations also fall under this category of experiences, which, although genuinely felt, present a false picture of reality. In these cases, intervention by another person, ideally a therapist or doctor, is necessary.

The studies on metacognition in schizophrenia have shown that the patients have difficulty thinking about both their own mental activities and the mental activities of others (14). For example, the majority of people with schizophrenia have difficulty with tasks that require integrating multiple pieces of evidence to reach a broader understanding of themselves and others (14). These deficits are described in such terms as Theory of Mind, emotion recognition, and Emotional Intelligence (14). In all these cases, either the representation of the owner (the representation “I”) or the representation of the subjective experience, although vivid for the subject, are delusive–they are not objectively true (7). The subject believes that he or she experiences a state with the content p, but p is false and what’s more, it cannot be falsified from the first-person perspective, because sometimes the subject simply does not know that he or she is deluded (15). The issue of the possible fall of the FPA is of major importance here. This problem concerns the cases where the patient experiences a state, which is for him/her real, but the doctor “knows better” the truth.

## Integrating metacognition in schizophrenia therapy

Regarding the role of FPA in metacognition, the treatment of schizophrenia presents particular challenges (16). People with schizophrenia often have trouble to form complex thoughts about themselves and others (16). The research results point out that positive symptoms are principally allied to social cognition, whereas negative symptoms impact both metacognition and social cognition (16). The severity of symptoms, such as hallucinations, delusions, and formal thought disorders, highlights the significant role, which impaired metacognition plays in schizophrenia. Impaired metacognition can namely lead to contradictory mental content and doubts about the reality of one’s experiences (16) and thus hinder the metacognitive therapy. Consequently, metacognition has become a rapidly expanding field of study as metacognitive deficits are a common feature of psychiatric disorders (16).

The research on metacognition initiated in the 80’s the development of metacognitive therapies – MCT (17) and psychometric metacognition assessment tools (18). According to the former, in Challenging Metacognitive Beliefs therapists help patients identify and question the first-person beliefs about themselves. In Attention Training Technique (ATT) patients practice focusing and shifting attention to reduce intrusive thoughts. And Cognitive Awareness Training improves awareness of one’s thought processes, enhancing healthier cognitive patterns (19). To evaluate metacognition such psychometric assessment tools as Metacognition Assessment Scale (MAS) are used, which allows for the evaluation of metacognitive processes through observed behaviour. Other tools are Metacognition Assessment Interview (MAI) and Metacognition Self-Assessment Scale (MSAS), which apply semi-structured interviews to measure domains like self-awareness and mentalizing. Self-administered scales, such as the Beck Cognitive Insight Scale and various Metacognition Questionnaires (MCQ) focus on specific metacognitive beliefs, anxiety, or dysfunctional thought processes across different age groups (17).

The right assessment of the metacognitive abilities of patients with schizophrenia is crucial for gaining a deeper understanding of the patient’s condition and developing effective treatment strategies (18). This evaluation is however often complicated because of the complex nature of schizophrenia and the cognitive impairments, which it causes, as for example a frequent lack of the insight (18). Schizophrenia can affect a subject’s ability to distinguish reality from delusion, comprehend questions, or retain information, which may undermine the reliability of empirical findings (18). The most significantly affected metacognitive processes in schizophrenia include forming accurate mental representations, performing cognitive tasks, and developing strategies to handle challenging mental situations (16). In addition to impaired metacognitive skills, also low social functioning has been observed (16). Metacognitive deficits are linked to the impairments in professional life, low self-esteem, and social anxiety (16). Patients in both early and advanced stages of schizophrenia, with the exception of addicts, had more difficulty in developing complex thoughts about themselves and others, but there was no significant difference between the groups in terms of metacognitive responses to psychological and social problems (20). Therefore, strengthening metacognitive skills also by respecting FPA in therapies can be fundamental in transforming cognitive abilities into functional life skills (Cf. 16).

## Metacognitive therapies and epistemic injustice

The importance of patients’ metacognitive abilities in the treatment of schizophrenia is often underlined (17). We assume that metacognitive therapies are successful, i.a. because they preserve FPA. At the same time therapies for mental disorders should be particularly sensitive to the risk of hurt to the FPA, because persons with psychiatric conditions are particularly vulnerable to testimonial and hermeneutical injustices, including the epistemic privileging of scientific and medical evidence, language, and concepts in discussions of psychiatric health and illness (21, 5-6). Considering the impact of epistemic injustice in therapy and treatment, it can be stated that mental disorders are more vulnerable to epistemic injustice than physical disorders (22). Epistemic injustice can be defined as “wronging particular knowers as knowers, for example by suppressing knowers testimony” (23, 13). The conflict between first-person reports and third-person testimony in such disabilities as schizophrenia can be interpreted in terms of epistemic injustice, because it applies exactly to the question of fallibility of the knowledge about one’s own mental states, and to situations in which the other person (doctor, therapist) has better knowledge about patient’s mental states. In other words an authoritative approach toward patients experiencing hallucinations or delusions can create a sense of epistemic injustice, where others claim better understanding of patient’s own experiences. An erosion or disruption of FPA can adversely impact the therapeutic process and further lower the patient’s self-esteem.

However, overemphasizing FPA at the expense of necessary medical oversight may reinforce patients’ maladaptive cognitions. Respecting FPA does not mean uncritically endorsing all patient beliefs. As mentioned above, people with schizophrenia often have difficulty to report and evaluate accurately their experiences, which may hinder full participation in the metacognitive therapies or cause false testimonies and thus influence the effect of therapy. The overestimation of FPA in cases of metacognitive disorders can lead to situations where patients, due to misinterpretations of the reality of their own experiences, reject a medical diagnosis (24). As Scrutton (25, p. 350) states: “In these cases, a medical perspective is often found to conflict with a meaningful one for the patients.” Although patients may recognize that their experiences, such as voice-hearing, have a pathological cause, they “tend to view their voices as more than just a bunch of symptoms that need fixing” (25, p. 350). Nevertheless, when skillfully conducted, metacognitive therapy allows the patient to navigate through the meanders of his or her mind, softly guiding them to explore alternative explanations or evidence-based treatments.

## Conclusion

Metacognitive therapies, when designed with sensitivity FPA, can fundamentally reshape schizophrenia treatment. To bridge theory and practice, clinicians should apply such strategies as structured metacognitive training programs, psychometric assessments, and guided discussions that respect patients’ experiences while providing evidence-based guidance. Tools like the Metacognition Assessment Scale (MAS) or Cognitive Insight Scales help clinicians create personalized treatment plans that meet each patient’s unique needs. Achieving this balance involves affirming the patient’s experiences, validating their perspective, and collaboratively addressing delusional content in a way that respects their autonomy, which helps to avoid the feeling of epistemic injustice.
